# Genome doubling enabled the expansion of yeast vesicle traffic pathways

**DOI:** 10.1038/s41598-022-15419-9

**Published:** 2022-07-02

**Authors:** Ramya Purkanti, Mukund Thattai

**Affiliations:** 1 Center for Integrative Genomics, Université de Lausanne, Lausanne, Switzerland; 2 Simons Centre for the Study of Living Machines, National Centre for Biological Sciences, Tata Institute of Fundamental Research, Bangalore, India

**Keywords:** Membrane trafficking, Evolution

## Abstract

Vesicle budding and fusion in eukaryotes depend on a suite of protein types, such as Arfs, Rabs, coats and SNAREs. Distinct paralogs of these proteins act at distinct intracellular locations, suggesting a link between gene duplication and the expansion of vesicle traffic pathways. Genome doubling, a common source of paralogous genes in fungi, provides an ideal setting in which to explore this link. Here we trace the fates of paralog doublets derived from the 100-Ma-old hybridization event that gave rise to the whole genome duplication clade of budding yeast. We find that paralog doublets involved in specific vesicle traffic functions and pathways are convergently retained across the entire clade. Vesicle coats and adaptors involved in secretory and early-endocytic pathways are retained as doublets, at rates several-fold higher than expected by chance. Proteins involved in later endocytic steps and intra-Golgi traffic, including the entire set of multi-subunit and coiled-coil tethers, have reverted to singletons. These patterns demonstrate that selection has acted to expand and diversify the yeast vesicle traffic apparatus, across species and time.

## Introduction

The eukaryotic vesicle traffic system is striking in its reliance on proteins encoded by large paralogous gene families. Distinct paralogs of Arf and Rab GTPases, vesicle coat proteins, cargo adaptors, and fusogenic SNAREs drive vesicle budding and fusion at distinct intracellular membranes^1^. The compositions of endomembrane organelles such as the endoplasmic reticulum, Golgi apparatus, and endosomes emerge dynamically, via the resulting loss and gain of vesicle cargo^2^. The organelle paralogy hypothesis posits that the generation of novel paralogs by gene duplication underlies the diversification of organelles over evolutionary time^3,4^. This hypothesis is supported by phylogenetic evidence^5,6^ and biophysical modeling^2^.

Vesicle traffic is regulated by function- and pathway-specific modules of interacting proteins^7^, so the expansion of traffic pathways is likely to depend on the duplication of multiple genes within a module^2–4^. However, the members of a module are typically dispersed across the genome^8^. There are two ways an entire module can be duplicated: stepwise, via successive segmental duplications of its constituent genes^9^; or simultaneously, via a genome doubling event^10^. Stepwise segmental duplication has given rise to certain multi-protein complexes involved in vesicle formation^9^, but these represent only a small proportion of vesicle traffic paralogs. Little is known about how the remaining gene duplications occurred. We hypothesized that genome doubling played a key role in this process. To test this idea we turned to the whole genome duplication (WGD) clade of budding yeast^11,12^, which includes the well-studied vesicle traffic model *Saccharomyces cerevisiae* (Fig. 1A). The WGD clade is the result of a 100-Ma-old interspecies hybridization event^13^ resulting in a cell in which each gene had two paralogs, one from each parental species.

Here we track the fates of these ancient paralog doublets to explore the link between gene duplication and vesicle traffic expansion. We show that genes encoding vesicle traffic proteins are significantly enriched among present-day doublets, compared to the genomic background (Fig. 1; Table 1). We find two key signatures of selection. First: doublets are lost or retained convergently across species (Fig. 1). Second: doublet retention is highly correlated with membership of specific protein modules (Figs. 2, 3). Vesicle coats and adaptors, and proteins that act along secretory and early-endocytic pathways, are retained as doublets. In contrast, tethers and SNAREs, and proteins that act in intra-Golgi transport, late endocytic steps and vacuolar dynamics, have reverted to singletons. Duplicate vesicle traffic modules have been described previously^14,15^. Our results go further, demonstrating that vesicle traffic expansion is among the most significant outcomes of the yeast WGD event, across all functional categories (Table 1). Though the yeast endomembrane system appears to be highly streamlined^16^, these patterns of paralog retention reveal layers of functional complexity sculpted by ongoing evolutionary processes.

## Results

**Figure 1:**
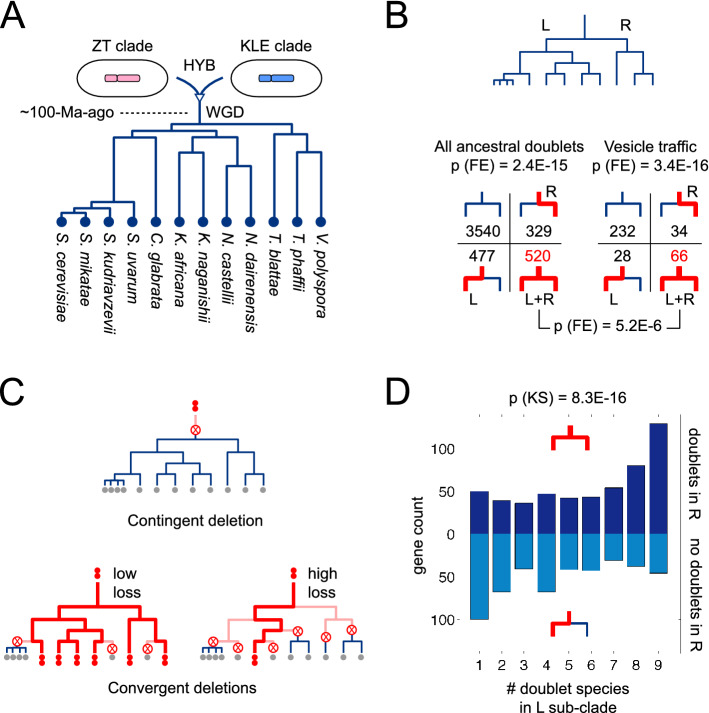
Convergent retention of paralog doublets after whole genome duplication. (**A**) The yeast WGD clade is descended from a hybridization between parents related to present-day members of the ZT and KLE clades, followed by a whole genome duplication event. See Fig. 4 for more details. Phylogenetic branch lengths are from Ref.^12^. (**B**) Top: Cladogram based on the phylogenetic tree from (**A**), indicating the left (L) and right (R) sub-clades. Bottom: Each paralog can be placed into one of four groups depending on whether it is present as a doublet in any member of the L or R sub-clades. We show the total number of paralogs in each of these groups, for all 4866 ancestral doublets (left) and the 360 ancestral doublets encoding vesicle traffic proteins (right). P-value calculations are described in the main text and “Methods”. (**C**) Paralog doublets (double red dots) can revert to singletons (single gray dots) by loss of one gene copy (crossed circle). Bold red lines: lineages where presence of a doublet can be inferred, since a doublet is present in a descendant. Light pink lines: lineages where a doublet was present but this cannot be inferred, since the doublet is lost in all descendants. Top: A single contingent deletion prior to the divergence of the clade. Bottom: Multiple convergent deletions on different branches, which can occur at low or high loss rates for different genes. (**D**) We test whether the presence or absence of doublets in the R sub-clade is predictive of the number of doublet species in the L sub-clade, conditioned on the doublet being present at the L-R branch point. The histograms in these two cases are significantly different, supporting the convergent scenario in (**C**).

### Ancestral paralog doublets in the yeast whole genome duplication clade

Species belonging to the yeast WGD clade are descended from an interspecies hybrid that underwent a genome doubling event^13^ (Figs. 1A, 4). The resulting cell initially contained two distinct copies of each gene (one from each parental species) which we term paralog doublets. We obtained high-confidence paralog assignments in 12 members of the yeast WGD clade from the Yeast Genome Order Browser^17^ (ygob.ucd.ie; “Methods”). This dataset uses conserved gene order (synteny) to identify paralogs derived via the founding hybridization event of the WGD clade, distinguishing these from other homologous copies of genes within each genome which may have arisen via earlier or later duplication events. For the remainder of our analysis we focus on the 4866 genes that we define as the ancestral paralog doublet set (“Methods”). Operationally, these are genes whose orthologs are found in the WGD clade as well as in the ZT and KLE clades that represent the closest living relatives of the two species involved in the original hybridization^13^. This definition has good specificity and sensitivity: with high confidence, the ancestral doublet set corresponds to genes present in two paralogous copies in the hybrid ancestor of the WGD clade. Over time, one or both copies may be lost^18^. Among ancestral doublets, 84% are present in at least one copy in every present-day WGD species, but only $\sim$ 10% are retained as doublets in any given species^19^. This suggests that the genes in this set play important roles in single copy, but that having paralog doublets is not typically advantageous^20,21^.

### Selection drives convergent retention of doublets across species

Paralog doublets may undergo neo-functionalization (where one copy takes on a non-ancestral function) or sub-functionalization (where the ancestral function is split between the two copies). Once either of these events occurs, selection would favour doublet retention^22–25^. We can detect signals of such selection by comparing evolutionary trajectories across multiple species. Since we are interested in long-term evolutionary patterns, we compared doublet occurrence in the two most distantly-related sub-clades of the WGD clade, which we refer to as the L and R sub-clades (Fig. 1A,B; “Methods”). If a paralog is present as a doublet in any member of a sub-clade, we can infer it was present as a doublet at the root of that sub-clade (Fig. 1C, bold red lines). If a paralog is present as a singleton in every member of a sub-clade, we cannot draw any conclusions: one copy may have been deleted early, prior to the divergence of the sub-clade; or deleted later, independently in every member of the sub-clade (Fig. 1C, light pink lines). Genes in the ancestral doublet set can be separated into four groups based on how they are retained in present-day species (Fig. 1B): those present as doublets in both sub-clades (L+R), those present as doublets in only one or the other sub-clade (L, R) or those present as singletons everywhere. Within the full gene set, doublet presence or absence is strongly correlated between the sub-clades ($\hbox {p} = 2.4\hbox {E}{-}15$, Fisher’s exact test; Fig. 1B, left).

The observed doublet retention correlation could be contingent on history, the result of very early losses prior to the divergence of the sub-clades (Fig. 1C, top); or it could be convergent, the result of multiple later losses within each sub-clade (Fig. 1C, bottom). In a contingent scenario, the pattern arises purely due to shared ancestry, and is not connected to gene function. In a convergent scenario, the pattern arises because homologous genes have similar loss rates across different lineages. In this case, we expect a correlation in doublet loss between the sub-clades, conditioned on the doublet still being present in their last common ancestor. We can distinguish between contingent and convergent deletions as follows (Fig. 1D). We pick a sub-clade, and only look at paralogs present as doublets in at least one member of that sub-clade; this enforces the condition that paralogs must be present as doublets in the last common ancestor of both sub-clades. For each paralog, we count the number of doublet species in this sub-clade. Finally, we split these paralogs into two groups, depending on whether or not they are present as doublets in some member of the other sub-clade. If the doublet enrichment pattern is purely contingent, doublet species counts should be statistically indistinguishable between these two groups. Instead we find (Fig. 1D) that they are highly distinct ($\hbox {p} = 8.3\hbox {E}{-}16$ for L sub-clade counts, $\hbox {p} = 4.2\hbox {E}{-}6$ for R sub-clade counts, Kolmogorov–Smirnov test). This rules out pure contingency, and implies convergent selection: some types of genes are more likely to be retained as doublets, and others more likely to revert to singletons, with losses occurring independently across species (Fig. 1C, bottom).

**Table 1:** Significantly enriched biological processes among L+R doublet members in *S. cerevisiae*, ranked by FDR. Processes related to vesicle traffic are in bold.

GO biological process	L+R doublet members		Fold enrichment	FDR
	Observed	Expected		
Biological regulation	354	236.12	1.5	4.54E−14
Regulation of cellular process	285	184.01	1.55	2.37E−11
Regulation of biological process	299	198.1	1.51	4.15E−11
Cellular process	659	592.29	1.11	2.17E−09
Cytoplasmic translation	59	20.07	2.94	3.20E−08
**Endocytosis**	45	14.32	**3.14**	1.03E−06
Cell communication	101	53.04	1.9	5.72E−06
Regulation of macromolecule metabolic process	196	129.8	1.51	7.13E−06
DNA recombination	3	27.81	0.11	6.56E−06
Regulation of biological quality	102	54.92	1.86	1.40E−05
**Vesicle-mediated transport**	96	50.7	**1.89**	1.45E−05
Regulation of metabolic process	202	138.83	1.46	3.30E−05

### Vesicle traffic genes have the highest fold enrichment among doublets

Our analysis is consistent with previous work showing that doublet retention is correlated with function^19,25–27^. To explore this connection in an unbiased manner, we examined the GO categories enriched among the 887 *S. cerevisiae* genes belonging to the L+R doublet set (“Methods”; Table 1; note that some L+R doublets are singletons in *S. cerevisiae*). Among the top GO categories ranked by statistical significance, ‘endocytosis’ has by far the highest fold enrichment (3.14$\times$), (with the super-category of ‘vesicle mediated transport’ also featuring on the list). Remarkably, this fold enrichment is even greater than that of the ribosomal genes typically presented as an extreme example of paralog retention^28^. The same result holds true across the entire WGD clade: even accounting for the genome-wide correlation of paralog doublets across species, vesicle traffic genes are significantly over-represented among the L+R set (66/360 = 0.18 compared to 520/4866 = 0.11; $\hbox {p} = 5.2\hbox {E}{-}6$, Fisher’s exact test; Fig. 1B).

**Figure 2:**
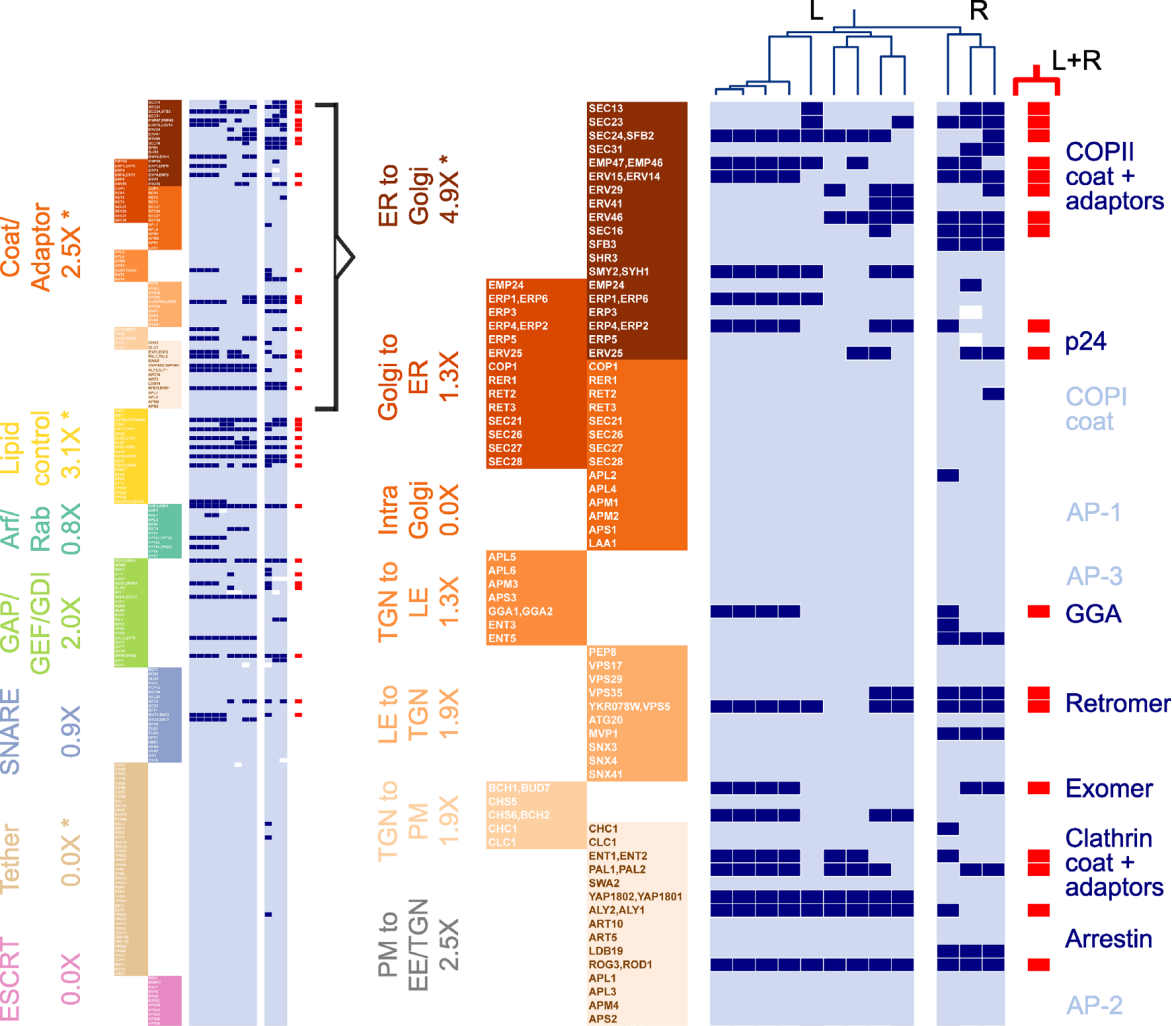
Function-specific and pathway-specific modules. We group 204 ancestral vesicle traffic genes into seven functional classes. We further sub-divide the Coat/Adaptor class into seven pathway-specific modules. *ER* endoplasmic reticulum, *PM* plasma membrane, *EE/TGN* early endosome/trans-Golgi network, *LE/PVE* late endosome/pre-vacuolar endosome. Note that some gene products can act at multiple locations. Genes are labeled by the names of the corresponding *S. cerevisiae* homologs. We track whether each gene is present as a doublet (dark blue), a singleton (light blue), or has been completely lost (white) in each of the 12 species of the WGD clade. This information is represented as a matrix: rows correspond to genes, columns correspond to species. The L and R sub-clades are separated for visual clarity. The Coat/Adaptor portion of the matrix is shown expanded on the right. Paralogs present as doublets in both the L and R sub-clades are highlighted in red. Under each class or module description, we show the enrichment of L+R doublets compared to the background expectation. P-value calculations are described in the main text and “Methods”; * represents statistically significant enrichment or depletion.

To further explore the role of function in doublet retention, we grouped genes into classes and modules. We define a module as a set of genes whose protein products act collectively to carry out specific vesicle traffic functions at specific cellular locations^1,7^. We manually assigned these genes to seven functional classes (Fig. 2, left) based on annotations from the *Saccharomyces* Genome Database^29^ (“Methods”). We further sub-divided vesicle coats and adaptors into seven modules based on the traffic pathways where they are active^30^ (Fig. 2, right). Out of 360 ancestral vesicle traffic doublets, we assigned 204 to classes and modules. Many of the remaining 156 genes play regulatory roles. Within each class or module, we asked how many paralogs were retained as doublets in both sub-clades (L+R doublets), and compared this to the expected number given the $\sim$ 18% (66/360) rate of L+R doublets among all vesicle traffic genes. Among functional classes, coat/adaptor genes and lipid control genes were enriched for L+R doublets; and tethers and ESCRT genes had no L+R doublets. Among coat/adaptor modules, ER to Golgi traffic genes and PM to EE/TGN traffic genes were enriched for L+R doublets; and intra-Golgi traffic genes had no L+R doublets. Only four of these cases were statistically significant (Fisher’s exact test, Benjamini–Hochberg correction, $\hbox {FDR} = 0.05$; “Methods”): ER to Golgi traffic (4.9$\times$ enrichment, raw $\hbox {p} = 6.6\hbox {E}{-}6$); coats/adaptors (2.5$\times$ enrichment, raw $\hbox {p} = 1.9\hbox {E}{-}4$); lipid control genes (3.1$\times$ enrichment, raw $\hbox {p} = 4.7\hbox {E}{-}3$); and tethers (0.0$\times$ enrichment, raw $\hbox {p} = 7.9\hbox {E}{-}3$).

### Doublets are retained in secretory and early-endocytic pathways

**Figure 3:**
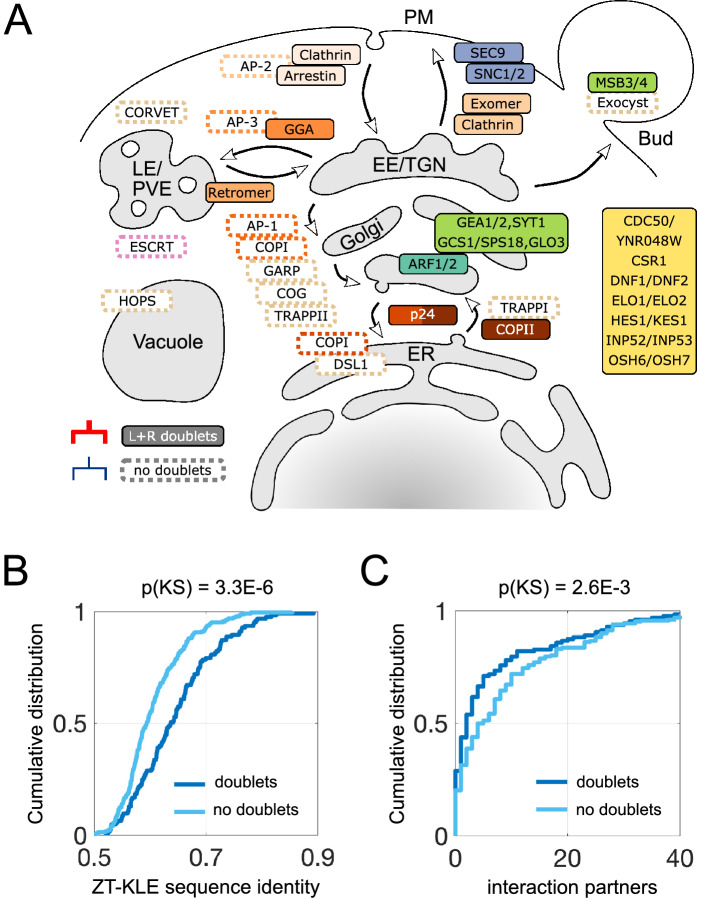
Landscape of vesicle traffic evolution in the WGD clade. (**A**) We show the site of action of proteins within the yeast vesicle traffic network. Filled boxes are proteins or complexes corresponding to genes present as L+R doublets. Dotted boxes show selected complexes for which the majority of proteins are present as singletons: the COPI coat and AP adaptors, tethers, and ESCRT. (**B,C**) We separate the 360 ancestral vesicle traffic doublets into two groups: those that are present as doublets in at least one species (dark blue) and those that are not doublets in any species (light blue). For each group, the curves show the cumulative distribution of two quantities. P-value calculations are described in the main text and “Methods”. (**B**) Inferred sequence identity between ZT and KLE orthologs (in *Z. rouxii* and *K. lactis*) corresponding to each ancestral doublet. (**C**) Number of physical interaction partners of the protein corresponding to each ancestral doublet.

We next considered the impact of paralogous modules in the context of the global yeast vesicle traffic system (Fig. 3A). Traffic pathways can be broadly classified into secretory components, from the ER via the Golgi and trans-Golgi network (TGN) to the plasma membrane (PM); and endocytic components, from the plasma membrane via early endosomes (EE) and late or pre-vacuolar endosomes (LE/PVE) to the vacuole. In *S. cerevisiae* the EE and TGN compartments appear to significantly overlap^16^, serving as transit points during both secretion and endocytosis. Given these and other ambiguities about the sites of action of vesicle traffic proteins, it is difficult to formulate and statistically test hypotheses about whether paralogs involved in specific pathways are more likely to be retained as doublets. Nevertheless, the following patterns are suggestive of general principles.

Every step of secretion from the ER to the plasma membrane involves paralogous L+R doublets. At the ER to Golgi step, multiple components of the COPII coat and its adaptors^31^, particularly the p24 complex^32^, are L+R doublets. Within the Golgi, the master regulator ARF1/ARF2 is an L+R doublet, along with many Arf modulators involved in anterograde traffic such as GEA1/GEA2^33^. At the TGN, cargo adaptors, including exomer which regulates TGN to PM export and GGA which regulates traffic to the PVE^30^, are L+R doublets, along with components of the clathrin coat. At the plasma membrane, the v/t-SNARE complex comprising SNC1/SNC2 and SEC9, which drives fusion of secretory vesicles to the PM, are L+R doublets. L+R doublets are also involved in early endocytic steps: early and intermediate clathrin coat proteins PAL1/PAL2 and ENT1/ENT2 are L+R doublets^34^, along with components of the endocytic regulator arrestin. Two components of retromer, an adaptor which regulates cargo flow from the LE to the TGN^30^, are L+R doublets.

In contrast to the above cases, several modules have completely reverted to singletons. These include: the COPI coat and all components of the AP adaptor complexes^30^; the entire set of coiled-coil and multi-subunit tethers^35^; and the ESCRT complex. With the exception of the exocyst complex and the AP-2 adaptor complex, which both act at the plasma membrane, the remaining singletons are all involved in retrograde Golgi traffic and late endocytic steps. The TRAPPI tether participates in ER-to-Golgi transport. The COPI and AP-1 coats, along with tethers GARP, COG, TRAPPII and DSL1, facilitate intra-Golgi cycling and Golgi-to-ER transport. The tethers CORVET and HOPS are involved in late endosomal and vacuolar dynamics, along with the ESCRT complex which remodels late endosomal membranes.

### Retained doublets have lower evolutionary rates and fewer protein interactions

Cross interactions between paralogous modules are common in newly formed yeast hybrids, even when parental species have diverged over 50 million years^36^. Tightly-interacting modules may be subject to dominant negative effects due to mutations in their paralogous partners, suggesting doublets involving highly interacting proteins are more likely to revert to singletons. However, it is also known that highly interacting proteins have lower evolutionary rates^37^, and in turn, lower evolutionary rates are correlated with lower rates of gene loss^38,39^. We sought to understand which of these two effects dominates.

As a proxy for the evolutionary rate of each ancestral doublet, we used the nucleotide sequence identity between the corresponding orthologs in present-day members of the ZT and KLE clades (“Methods”; this avoids the confounding effect of evolutionary rate variation between singletons and doublets in WGD clade species). We imputed a physical interaction network among the proteins encoded by ancestral doublets, using present-day interaction data for the corresponding proteins in *S. cerevisiae*^40^ (“Methods”). We separated all 360 ancestral vesicle traffic doublets into two groups: those present as pure singletons across all present-day species, and those present as doublets in at least one present-day species. We then compared the distributions of evolutionary rates and protein interaction degrees between these two groups (Fig. 3B,C). We find that doublet retention is strongly associated with lower evolutionary rates (higher sequence identity; $\hbox {p} = 3.3\hbox {E}{-}6$, Kolmogorov–Smirnov test; Fig. 3B). In contrast, doublet retention is only weakly associated with fewer protein interactions ($\hbox {p} = 2.6\hbox {E}{-}3$, Kolmogorov–Smirnov test; Fig. 3C). This is consistent with prior observations: cross-interactions after gene duplication are weakly correlated with fitness, due to compensatory mechanisms such as expression attenuation^21^; in contrast, low evolutionary rates are strongly correlated with low gene loss because functionally important genes tend to be under purifying selection^39^. Taken together, these data reinforce our finding that doublet retention is driven by selection for specific function.

## Discussion

Genome doubling is a recurring theme in eukaryotic evolution^20^. These events provide many opportunities for selection to act, and can reveal evolutionary pressures that are invisible under normal circumstances. In this study we have taken advantage of an ancient genome doubling event to rigorously demonstrate signatures of such selection on the yeast vesicle traffic system.

**Figure 4:**
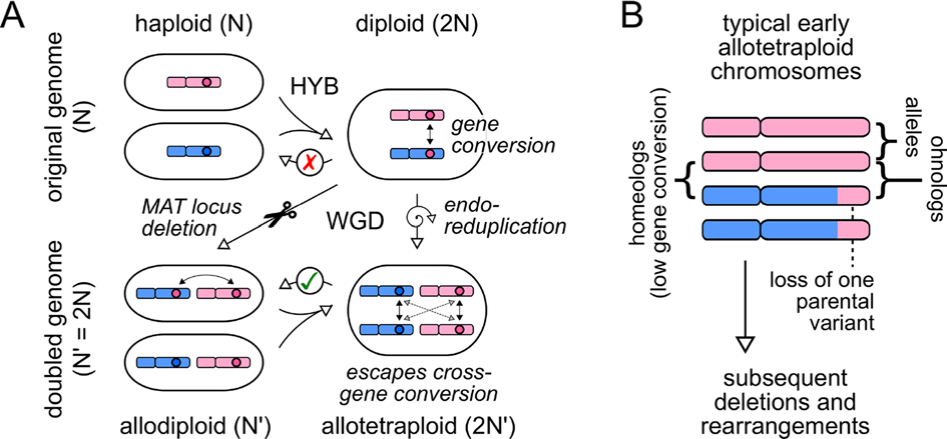
Homeologs and ohnologs in interspecies hybrids. (**A**) Routes to genome doubling in an interspecies hybrid. Pre-WGD hybrids are typically sterile (red cross) but WGD restores fertility (green tick). Allelic pairs in the pre-WGD cell become paralog pairs in the post-WGD cell. The WGD step can occur in one of two ways^41^: deletion of the MAT locus (diagonal arrow), which converts the pre-WGD diploid to a genome-doubled allodiploid (effectively a mating-competent haploid)^42,43^; or endo-reduplication (vertical arrow), which converts the pre-WGD diploid to a genome-doubled allotetraploid (effectively a fertile diploid)^44^. In the allodiploid, gene conversion can lead to loss of one parental variant of a paralog pair. In the allotetraploid, gene conversion occurs predominantly between the WGD-derived alleles rather than the hybridization-derived paralogs. (**B**) Typical organization of allotetraploid chromosomes soon after WGD. This schematic is based on the genome of the lager brewing yeast *S. pastorianus*, a 500-y-old interspecies hybrid^45,46^. All genes are present as allelic pairs, most of which are completely homozygous due to gene conversion or introgression. Homeologs are paralog pairs in which one variant is inherited from each parent. Ohnologs are paralog pairs in which one parental variant has been replaced by the other, by meiotic recombination in a pre-WGD hybrid diploid or by subsequent gene conversion.

Interspecies hybridization is a common route to genome doubling among fungi^47–49^. Interspecies diploids are typically sterile, since mismatches between homologous chromosomes stall meiosis; genome doubling spontaneously restores fertility in such hybrids, by creating an allotetraploid cell with two identical copies of each chromosome^41,43^ (Fig. 4A). The alleles of the original hybrid diploid become paralog doublets of the allotetraploid. Paralogs are always at risk of being lost due to gene conversion, which occurs when homologous template sequences are used to repair double-strand breaks^50^. Newly-formed hybrid allotetraploid genomes typically contain both homeologs (pairs derived from both parents) and ohnologs (pairs tracing to a single parent, due to pre-WGD gene conversion)^51,52^ (Fig 4B). Gene conversion rapidly erases variations between ohnologs^10,42,44^ but spares the more diverged homeologs, since double-strand break repair in allotetraploids uses alleles as templates^45,46^ (Fig. 4B). Homeologs thus have more opportunities for neo-functionalization or sub-functionalization, compared to ohnologs. Consistent with this, the majority of paralogs retained as doublets in present-day *S. cerevisiae* (63%), including most vesicle traffic doublets (66%), are homeologs (“Methods”).

Paralogous modules have been retained in the yeast vesicle traffic system at rates much higher than expected by chance, 100 million years after they arose by hybridization. Even more striking is the clear convergence of evolutionary trajectories across diverse yeast species, indicating common selection pressures operating on the vesicle traffic system over time and across ecological contexts. It is likely that there are multiple mechanisms by which paralog doublets confer a selective advantage. For paralogs with highly overlapping functions, gene dosage may act to increase the capacity of vesicle traffic pathways^53^ (EMP46/EMP47^54^; BCH1/BUD7^55^; ENT1/ENT2^56^). Paralogs that are differentially expressed or regulated may help cells to tune their vesicle traffic pathways under different conditions^57^( RCR1/RCR2^58^; ERV14/ERV15 and SEC24/SFB2^59,60^; ART1/ART2^61^; EMP46/EMP47 and SDS23/SDS24^62,63^; ARF1/ARF2^64^). Paralogs may have distinct interaction partners (Pkh1/Pkh2^65^), distinct cellular locations (Gea1/Gea2^66^; Rcr1/Rcr2^58^), or other types of distinct properties (ALY1/ALY2^67^; PAL1/PAL2^68^; GGA1/GGA2^69,70^; HES1/KES1 and OSH6/OSH7^71^). In all these ways, the presence of doublets potentially increases the versatility of the vesicle traffic system.

The last eukaryotic common ancestor (LECA) had distinct, homologous versions of Arfs, Rabs, coats, and SNAREs which operated along distinct trafficking pathways. These same protein families comprise organelle-specific modules in all present-day eukaryotes. Yet the patterns of gene duplication subsequent to LECA appear to vary across lineages. The large family of tethers, essential for vesicle fusion, is comparable in size to other protein classes involved in vesicle traffic^35^. In yeast, tethers seem to have expanded by sporadic recruitment rather than by gene duplication^72^. This pattern finds echoes in our analysis: strikingly, all 47 tethers in our dataset have reverted to singletons in nearly every WGD species. However, this pattern does not hold outside of fungi: metazoans have two paralogs of the HOPS and CORVET components VPS16 and VPS33^73^, while ciliates have paralogous copies of multiple CORVET components^72^. More broadly, the pre-LECA and post-LECA phases of gene family expansion appear to be fundamentally different in character. Though we find that many paralogs derived from genome doubling have been retained within the yeast vesicle traffic system, there are nearly no examples of paralogs operating at highly distinct sub-cellular locations. This suggests that the architecture of vesicle traffic in present-day eukaryotes is tightly constrained, and that the genome doubling route we have explored is distinct from more ancient duplication routes. It is likely the major vesicle traffic gene families were generated during an earlier, more dynamic and less constrained phase of eukaryotic evolution.

## Methods

### Ortholog assignments in pre-WGD and post-WGD species

We downloaded synteny-based ortholog assignments and paralog pair assignments from the Yeast Genome Order Browser^17^ (YGOB Version 7; ygob.ucd.ie). This dataset covers 20 species: 12 within the yeast WGD clade, which we split into two sub-clades for further analysis (L sub-clade: *S. cerevisiae*, *S. mikatae*, *S. kudriavzevii*, *S. uvarum*, *C. glabrata*, *K. africana*, *K. naganishii*, *N. castellii*, *N. dairenensis*; R sub-clade: *T. blattae*, *T. phaffii*, *V. polyspora*); and 8 pre-WGD species comprising the ZT clade (*Z. rouxii*, *T. delbrueckii*) and the KLE clade (*K. lactis*, *E. gossypii*, *E. cymbalariae*, *L. kluyveri*, *L. thermotolerans*, *L. waltii*). The WGD clade is descended from an interspecies hybridization between two species whose closest living relatives are inferred to belong to the ZT clade and the KLE clade, respectively^13^. A total of 14101 orthologs are present across all 20 species in the dataset. A subset of 11059 orthologs are found within the WGD clade.

### Defining the ancestral paralog doublet set

We are interested in orthologs that were present as paralog doublets immediately following the original interspecies hybridization. By definition, one copy of each such gene is inherited from each parent. However, we do not know the true genetic complement of the parental species, only that of their closest living relatives. Operationally, we define the set of ancestral doublets as the set of 4866 genes found across the ZT, KLE and WGD clades. This definition has good specificity (3891 genes in this set are present in every member of the ZT and KLE clades, and were therefore likely to be inherited as doublets in the WGD ancestor) and good sensitivity (only 48 out of the 1374 genes present as doublets in any present-day WGD species are are missing from this set). 4075 out of 4866 genes are present in at least one copy in every present-day WGD species. The full list of ancestral doublets is provided in Supplementary Table S1.

### Classifying doublets as homeologs and ohnologs

The time of duplication of paralog doublets has been estimated using phylogenetic methods, as described in^13^. We obtained supporting data associated with this study. The duplication event is assigned to a branch of a species tree spanning the KLE, ZT and WGD clades, as defined in Fig. 1 of Ref.^13^. Each doublet is associated with two inferred duplication branches, based on the phylogenetic trees seeded by each doublet member. In the event that the two branches do not match, we retained the branch with higher support. Those with support below 0.95 were not considered. In this way, we could assign the duplication branch for 60% (377/620) of *S. cerevisiae* paralog doublets (Supplementary Table S1). Homeologs correspond to branches $\le$ n4 (duplicated prior to WGD) and ohnologs correspond to branches $\ge$ n5 (duplicated at or after WGD). 63% (239/377) of all doublets and 66% (31/47) of vesicle traffic doublets are homeologs. Duplication branches are listed in Supplementary Table S1.

### Annotation of vesicle traffic genes

We assigned genes to functional categories based on annotations of their *S. cerevisiae* homologs. 426 *S. cerevisiae* genes are annotated with the Gene Ontology term GO:0016192 ‘vesicle-mediated transport’^74^ (implemented via PANTHER Version 16.0; pantherdb.org). To these we added 17 genes whose paralogs were already part of the set. This resulted in 443 genes (323 singletons and 60 doublets in *S. cerevisiae*) of which 360 are present in the ancestral paralog doublet set (300 singletons and 60 doublets in *S. cerevisiae*). We used annotations from the *Saccharomyces* Genome Database^29^ (yeastgenome.org) to assign 236 out of 443 genes (204 out of 360 ancestral vesicle traffic doublets) to seven functional classes: Coat/Adaptor; Lipid control; Arf/Rab; GAP/GEF/GDI; SNARE; Tether; and ESCRT. We sub-divided the Coat/Adaptor class into seven pathway-specific modules^30^: ER to Golgi (COPII, p24); Golgi to ER (COPI, p24); Intra Golgi (clathrin, AP-1, COPI); TGN to LE (AP-3, GGA, epsin); LE to TGN (retromer, nexin); TGN to PM (clathrin, exomer); and PM to EE/TGN (clathrin, AP-2, arrestin). Some Coat/Adaptor genes are active in more than one pathway. Annotations of vesicle traffic genes are provided in Supplementary Table S1.

### Enrichment analysis and statistical tests

We identified 887 genes in *S. cerevisiae* that corresponded to L+R doublets. On this gene list we carried out an overrepresentation test using PANTHER (PANTHER Version 17.0; pantherdb.org)^74^ for ‘GO Biological Process Complete’ functional categories, with the yeast genome as the background. We sorted hits by the False Discovery Rate (Benjamini–Hochberg procedure, overall $\hbox {FDR}<0.05$). The top 12 hits are shown in Table 1, the top 20 hits and full details shown in Supplementary Table S2. We performed other enrichment analyses (Figs. 1B, 2) using the two-tailed Fisher’s exact test on $2 \times 2$ contingency tables. When testing for enrichment among the 14 vesicle traffic gene classes/modules (Fig. 2), we additionally applied the Benjamini–Hochberg correction for multiple hypothesis testing with a false discovery rate $\alpha$ = 0.05, to determine the significance threshold. For comparing between distributions (Figs. 1D, 3B,C) we used the Kolmogorov–Smirnov (KS) test. Note that the KS test reports a conservative p-value when applied to discrete distributions. Data and p-values for each test are provided in Supplementary Table S3.

### Protein interaction analysis

The 360 ancestral vesicle traffic genes correspond to 420 *S. cerevisiae* genes (300 singletons and 60 doublets). For these genes we obtained the protein–protein interaction network from the STRING database^40^ (string-db.org), filtering for the physical sub-network at medium confidence, with experiments and databases as interaction data sources. For genes present as doublets, we assumed an interaction between a pair of ancestral genes if there was an interaction between any of their paralogs, as would be expected based on a sub-functionalization scenario. Note that this systematically increases the inferred interaction degree of doublets. Even so, we find that doublets overall have fewer interactions than singletons (Fig. 3C). Interaction data are provided in Supplementary Table S1.

### Evolutionary rate analysis

To estimate the evolutionary rate of ancestral paralog doublets, we examined the sequence identity between the corresponding orthologs in *Z. rouxii* and *K. lactis* (representative species for the ZT and KLE clade). We used the YGOB database (Version 7; ygob.ucd.ie) to assign orthologs. Of the 4866 ancestral doublets, 4585 had orthologs in *Z. rouxii* and *K. lactis*, of which 4490 sequences were protein-coding gene pairs. We aligned the corresponding gene pairs and computed the nucleotide sequence identity. The mean identity was $0.644\pm 0.003$ for ancestral doublets with at least one present-day doublet, and $0.616\pm 0.001$ for ancestral doublets with no present-day doublets (mean ± SEM). Sequence identity values are provided in Supplementary Table S1.

## Supplementary Information

Supplementary Tables.

## Data Availability

All data generated or analysed during this study are included in this published article and its Supplementary Information files.
